# Epidemiological Characteristics and Spatial-Temporal Clusters of Mumps in Shandong Province, China, 2005–2014

**DOI:** 10.1038/srep46328

**Published:** 2017-04-11

**Authors:** Runzi Li, Shenghui Cheng, Cheng Luo, Shannon Rutherford, Jin Cao, Qinqin Xu, Xiaodong Liu, Yanxun Liu, Fuzhong Xue, Qing Xu, Xiujun Li

**Affiliations:** 1 Department of Epidemiology and Biostatistics, School of Public Health, Shandong University, Jinan 250012, Shandong, China; 2 Visual Analytics and Imaging Lab, Computer Science Department, Stony Brook University, New York 10024, America; 3 School of Medicine & Centre for Environment and Population Health, Griffith University, Queensland 4218, Australia; 4 Shandong Center for Disease Control and Prevention, Jinan 250012, Shandong, China

## Abstract

Mumps presents a serious threat to public health in China. We conducted a descriptive analysis to identify the epidemiological characteristics of mumps in Shandong Province. Spatial autocorrelation and space-time scan analyses were utilized to detect spatial-temporal clusters. From 2005 to 2014, 115745 mumps cases were reported in Shandong, with an average male-to-female ratio of 1.94. Mumps occurred mostly in spring (32.17% of all reported cases) and in children aged 5 to 9 (40.79% of all reported cases). The Moran’s I test was significant and local indicators of spatial autocorrelation (LISA) analysis revealed significant spatial clusters with high incidence. The results showed that the mid-west of Shandong Province and some coastal regions (Qingdao City and Weihai City) were high-risk areas, particularly in the center of the Jining City and the junction of Dongying City, Binzhou City and Zibo City. The results could assist local and national public health agencies in formulating better public health strategic planning and resource allocation.

As an acute respiratory and viral member in the paramyxovirus family, mumps is identified by the symptom–a nonspecific precursor of fever, accompanied with swollen and painful glands in the neck^1^. Although this infection in most cases is mild, numerous clinical consequences may occur with complications, such as deafness, mastitis, pancreatitis, oophoritis, and meningitis (up to 15% of cases)^2^,^3^. In the long run, mumps is one of the major causes of children’s acquired sensorineural deafness, with an incidence of about 5/100 000^4^. Most countries have introduced mumps vaccine primarily for adolescents over the past decades. For example, in China, live attenuated mumps vaccine was firstly introduced in the 1990s, and has been included in the national routine program for immunization since 2008^5^,^6^. Children aged 18–24 months routinely can receive one dose of measles-mumps-rubella vaccine (MMR) without charge^7^. However, several mumps outbreaks were witnessed in China in 2012 and in other countries such as France in 2013, Ireland in 2008, and the UK in 2000 and 2005^8^,^9^,^10^,^11^,^12^. More importantly, an increasing trend was observed in China from 2005–2010^13^ when the annual incidence rate of mumps was 20 per 100,000 to an increase in 30 per 100,000 during 2011–2012. This data suggests that mumps remains a serious issue to public health.

The spatial distribution data indicates regional differences for mumps in China^14^,^15^,^16^. The spatial analysis methods have been frequently utilized to describe epidemiological characteristics and analyze the clusters for some important infectious diseases. For example, spatial analysis has been conducted for the geographical distribution of hemorrhagic fever with renal syndrome^17^. Other similar techniques like geographic information systems have been applied in Suriname to characterize the epidemic pattern of dengue fever and detect clusters^18^.

Global climate change has enhanced the spread of infectious diseases^19^, which requires clarification of the characteristics of mumps in spatial and temporal domains. However, prior studies on the spatial epidemiology of mumps in China have been limited with only three specific regions, including Shanxi Province (a plateau province of North China), Yiwu city (Zhejiang Province, a coastal province between East and South China) and Gansu Province (Northwest China) were examined^14^,^15^,^16^. We aimed to investigate the spatial epidemiology of mumps in Shandong Province, a coastal province of North-Central China.

A spatiotemporal analysis of mumps could support health departments in formulating regional prevention and control strategies^20^,^21^. Traditional statistical analysis without displays or graphics limits its utility, particularly for advising policy makers on priority problems. Visualization encodes data as graphics or images, and is able to display the data in a more intuitive way. Thus, our spatial analysis is based on the visualization generated by GIS to show mumps distribution. To distinguish spatiotemporal clusters of mumps, spatial autocorrelation analysis and spatial cluster analysis were conducted. Based on the strategies above, we could characterize geographic distribution patterns of mumps in Shandong Province during 2005–2014.

## Results

From 2005 to 2014, 115745 mumps cases were reported in Shandong Province–39312 females and 76433 males. The characteristics of mumps cases from 2005 to 2014 are shown in Table 1. Children aged 5 to 9 accounted for the majority of cases (40.79%). The average male-to-female ratio was 1.94 (1.89 in 2005, 2.05 in 2007, 2 in 2009, 2.04 in 2011, and 1.78 in 2014). Mumps occurred mostly in spring–32.17% (37231 cases) of all reported cases.

**Figure 1:**
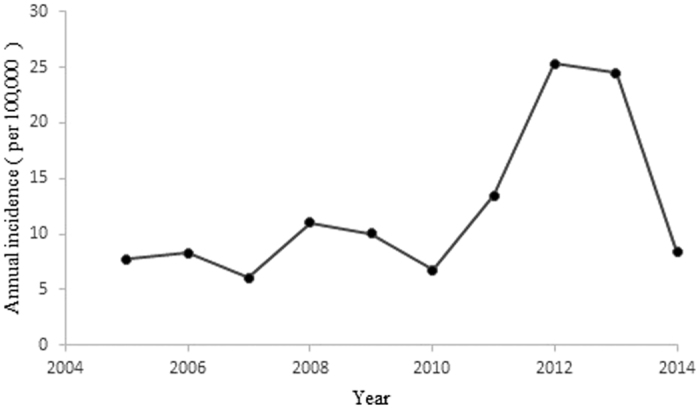
The annual incidences of mumps in Shandong Province, China, 2005–2014. The figure shows the annual incidences of mumps in Shandong Province from 2005 to 2014.

**Figure 2:**
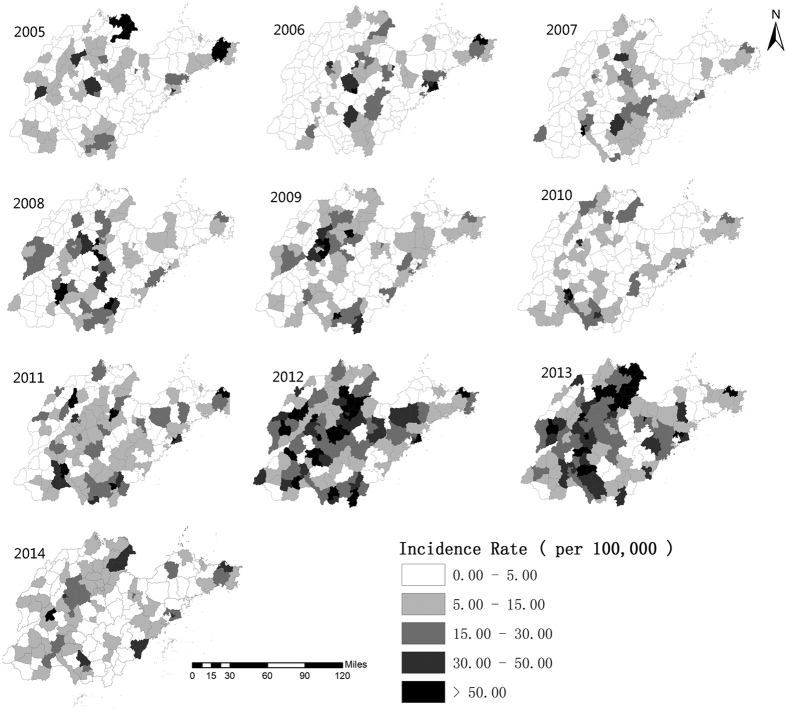
Geographical distribution of the incidence rates of mumps in Shandong Province, China at the county level from 2005–2014. The incidence rates of mumps are colored with different colors in the map using the software ArcGIS10.2 (https://www.arcgis.com/features/index.html, ESRI, Inc., Redlands, CA, USA).

**Figure 3:**
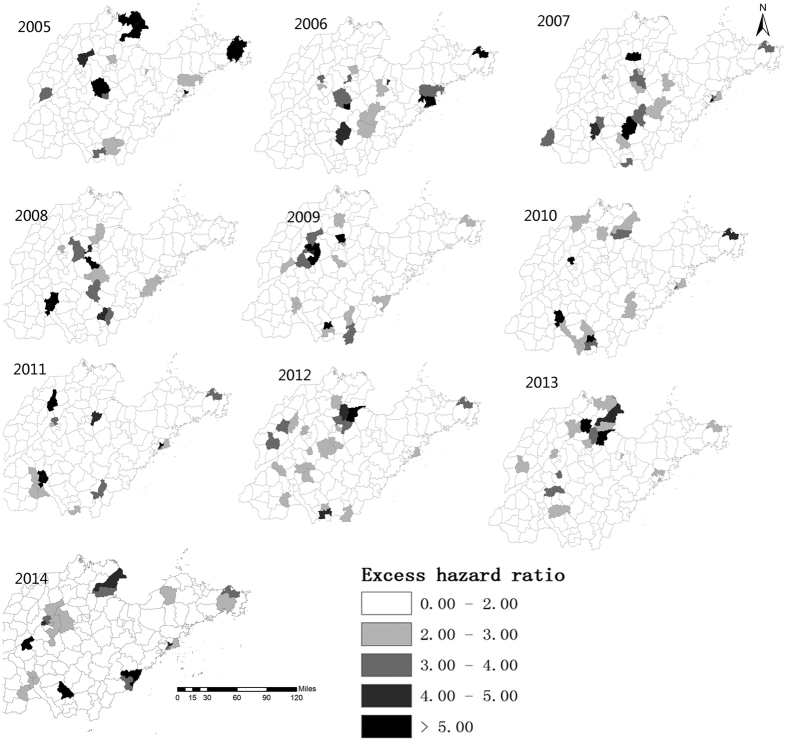
The hazard ratio of mumps in Shandong Province, China at the county level from 2005–2014. The hazard ratios of mumps are colored with different colors in the map using the software ArcGIS10.2 (https://www.arcgis.com/features/index.html, ESRI, Inc., Redlands, CA, USA).

From 2005 to 2014, the annual incidences of mumps in Shandong Province ranged from 6.10 (in 2007) to 25.34 per 100,000 (in 2012) (Fig. 1). Figure 2 illustrates the heterogeneity of geographical distribution of mumps. The top three highest incidence regions were Wendeng (Yantai City), Kenli (Dongying City), and Hekou (Dongying City) in 2005. In 2010 these three regions changed to Shizhong (Zaozhuang City), Shizhong (Jining City), and Rencheng (Jining City). The top three highest incidence regions in 2014 were Pingyin (Jinan City), Donggang (Rizhao City), and Shanting (Zaozhuang City). Figure 3 illustrates the hazard ratio of mumps in Shandong Province.

**Figure 4:**
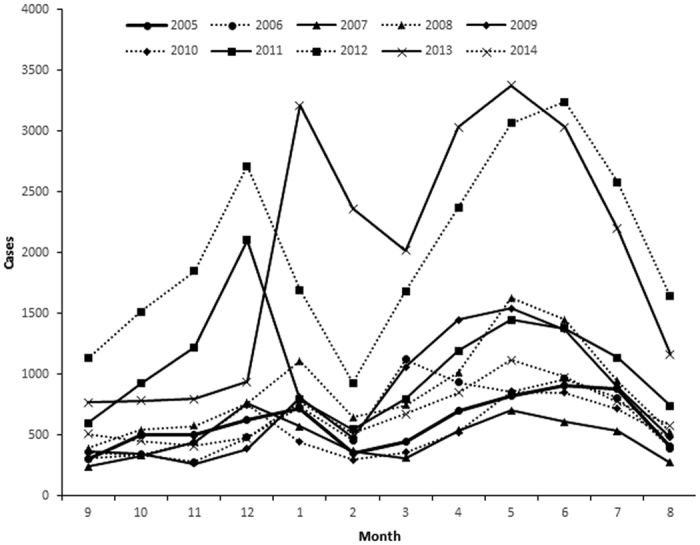
Monthly cases of mumps disease in Shandong Province, China from 2005–2014. The figure shows the mumps cases by month from 2005 to 2014.

Figure 4 shows mumps’ cases by month from 2005 to 2014. Mumps cases were highly influenced by season. The seasonal trend of mumps was similar among different years–a small peak in December-January, a big increase from May- June, with similar peaks in winter and a joint period across spring and summer.

Table 2 shows the global spatial autocorrelation of mumps. The Moran’s I was significant for every year. It ranged from 0.15 in 2005 to 0.46 in 2006 (p < 0.005), implying the existence of the heterogeneous distribution and high spatial dependency in Shandong Province.

From 2005 to 2014, statistically significant spatial clusters of mumps were found using the local spatial autocorrelation test (Table 3). The hotspots were mainly distributed in Lixia and Shizhong in Jinan City, Shinan, Shibei, Sifang, Laoshan, Licang and Chengyang in Qingdao City, Linzi, Gaoxinjishukaifa and Gaoqing in Zibo City, Dongying in Dongying City, Shizhong in Jining City, and Boxing in Binzhou City from 2005 to 2014.

**Figure 5:**
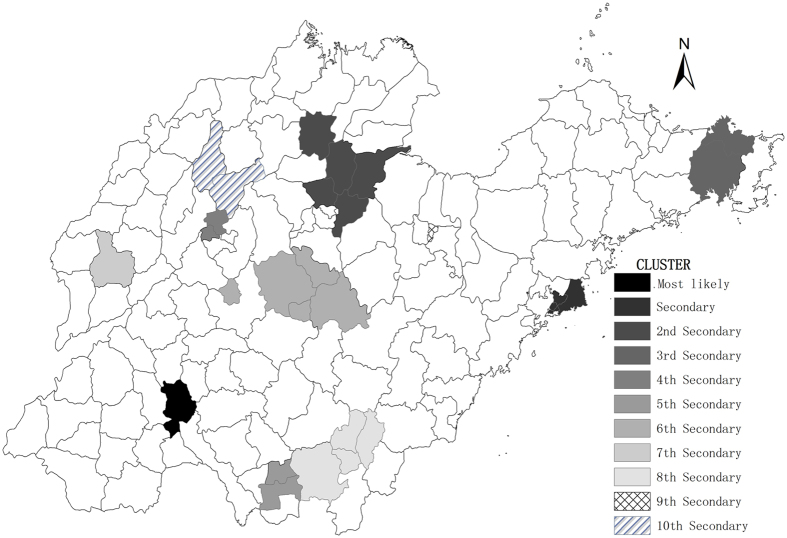
Spatial clusters of mumps in Shandong Province, China from 2005–2014. The space scan proposed by Kulldorff is integrated in SaTScan^TM^ v9.4 (http://www.satscan.org/) and clusters are depicted on the map using the software ArcGIS10.2 (https://www.arcgis.com/features/index.html, ESRI, Inc., Redlands, CA, USA).

The space scan analysis of mumps indicated that the distribution was not random in Shandong Province. There were one most likely statistically significant cluster and ten statistically significant secondary clusters for high incidence of mumps identified by spatial scan statistic. The most likely clusters were distributed in Shizhong and Rencheng in Jining City during 2005–2014 (RR = 3.38, *p* < 0.0001, radius = 4.88), with 8992 observed cases and 2805.17 expected cases. The clusters are depicted on the map in Fig. 5.

**Figure 6:**
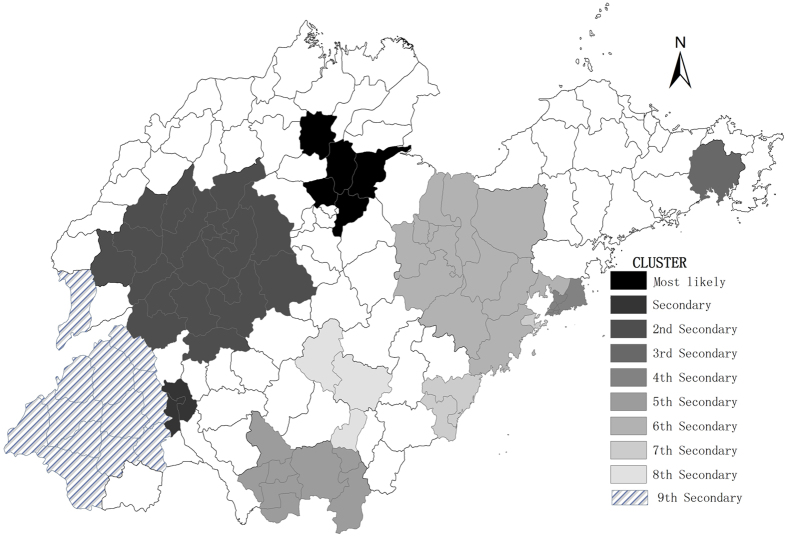
Spatial-temporal clusters of mumps in Shandong Province, China from 2005–2014. The space-time scan proposed by Kulldorff is integrated in SaTScan^TM^ v9.4 (http://www.satscan.org/) and clusters are depicted on the map using the software ArcGIS10.2 (https://www.arcgis.com/features/index.html, ESRI, Inc., Redlands, CA, USA).

In this section, we used space-time analysis to identify clusters from 2005–2014. This analysis showed that mumps was not distributed randomly in space and time. The spatial-temporal scan statistics indicated that there was one most likely statistically significant cluster consisting of five adjacent counties–Boxing in Binzhou City, Guangrao in Dongying City, Huantai in Zibo City, Bincheng in Binzhou City, and Linzi in Zibo City for the year 2012–2013, (RR = 9.48, *p* < 0.001),with 6333 observed cases and 699.82 expected cases. The results are listed in Table 4, and depicted on the map in Fig. 6.

## Discussion

In the past decades, many countries have introduced mumps vaccine, however, several outbreaks have been witnessed in many countries. Mumps remains a severe global public health problem in numerous developing countries. There are more male patients than female. Prior studies have shown that males have a higher morbidity rate than females^6^. During 2005–2014, mumps cases aged 5–9 years old increased-more than 40% of the overall cases. This is consistent with the epidemiologic characteristics of mumps^1^. Some epidemiological characteristics differ region by region. For example, spring is the high-risk season in Taiwan^22^; summer is high-occurrence season for Guangzhou Province, China^6^; in Shandong Province, mumps occurs mostly in late spring, early summer and winter. These differences indicate that the local geography and meteorological factors probably impact mumps’ incidence and transmission.

In this study, spatial autocorrelation analysis was conducted in Shandong Province, China. Local spatial autocorrelation analysis detected spatial clusters of mumps with high-high pattern. The clusters with the high-high pattern were recognized as the hotspots. There was a decreasing trend in the Moran’s I from 2005 to 2014. This may be connected with the effective response to mumps.

The aims from the spatial and spatiotemporal analyses are different. In essence, the former analysis aims to identify areas with raised incidence throughout the full period, while the latter analysis aims to identify areas with raised incidence at specific periods. In this study, we utilized these two methods for data analysis, then made the comparison. The clusters obtained from these two methods lead to similar significant high-risk spatial clusters. It is confirmed that the mumps is not randomly distributed.

The results indicated that the mid-west of Shandong and some coastal regions were high-risk areas, especially in the center of Jining City and the junction of Dongying City, Binzhou City and Zibo City. In the mid-west of Shandong, these areas have poorer living conditions and sanitation compared to that in eastern Shandong. Socio-economic status may contribute to high incidence and cluster. At the border region and coastal region, immigrant foreigners lead to population growth, so that more social mobility and the resources for public health are demanding. However, the current state can not meet these requirements, making it easier to become high-risk region.

The space-time clusters were found in the year 2012–2013 which might be related to vaccine and apparent epidemic cycle of mumps. From 2005 to 2011, there were no significant differences in the annual incidences of mumps in Shandong Province, but a sharp rise in incidence occurred in 2012. The growth slowed down in 2013 and 2014 which might be related to the supplemental immunization. Since May 1, 2013, six-year-old children receive free vaccinations of measles and mumps, instead of the vaccine of measles^23^. This is able to increase vaccine immunization rates of mumps and reduce incidence.

The other similar studies such as the south in Shanxi Province, the Hexi Corridor (West Gansu Province) in Gansu Province and the Jiangdong street (Central and East Yiwu city) in Yiwu city of Zhejiang Province were existing high-risk areas^14^,^15^,^16^. The results in Shandong Province indicated that the mid-west of Shandong and some coastal regions were high-risk areas. All these high-risk areas in different studies generally existed same problems such as large migrant floating population, relatively high population densities, poor quality dwelling and living conditions, etc. It may be the cause of clusters. The decision-maker of government should pay more attention to the high risk areas in China.

Our research has some advantages in terms of mumps’ study and prevention. First of all, our study is based on the county-level data and the data is large and accurate. This allows analysts to flexibly explore the cluster. Second, a number of studies have been done on the spatial epidemiology of multiple communicable diseases, but regarding to mumps, few research can be found. Our study focuses on the spatial and temporal epidemiology of mumps in Shandong Province, which has not been reported yet. We confirm that this study is able to assist public health officials in mumps controlling, epidemics prediction, medical service sources disposition etc.

Meanwhile, our research has some limitations. First, some missing reporting cases existed, which might lead to systematic bias^24^. However, this happens quite rarely and non-response rates every month remain stable^25^, therefore this will not impact the spread trend of the disease. Second, the clusters of mumps might be affected by a range of factors including migration, local health conditions, economic conditions and local geography^26^, but this information was not collected for the cases and so identifying the reasons for the clusters is difficult. Hence future research should focus on the collection and utility of other relevant factors such as economic conditions and the environment factors in the analysis.

## Conclusion

This study explored the spatial epidemiological features of mumps from 2005 to 2014 in Shandong Province, China. It shows that spatial distribution patterns are aggregated at county level and the clusters vary significantly year by year. We propose that a real time spatial-temporal surveillance system is necessary, in order to find high risk areas, follow the epidemic trend and provide better prevention.

## Materials and Methods

### Study areas

**Figure 7:**
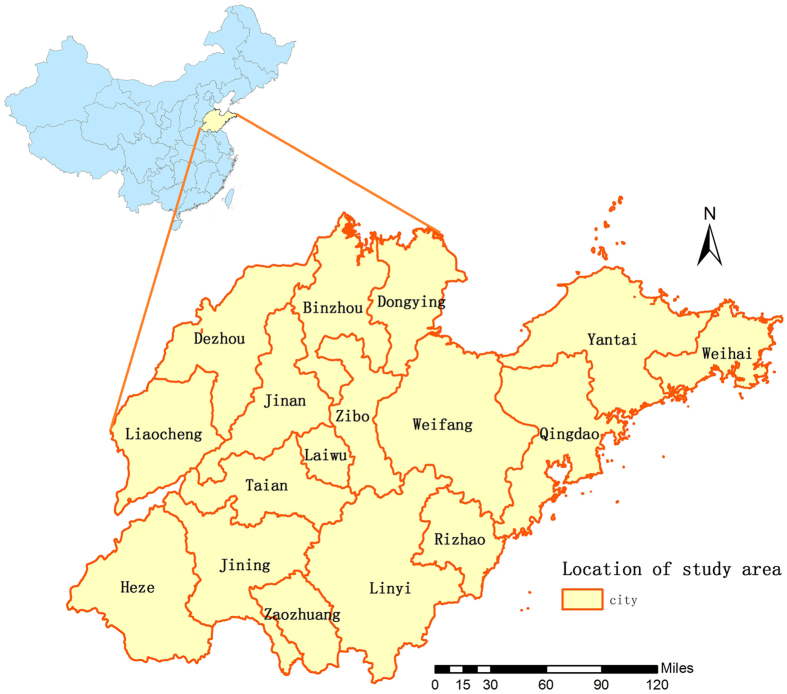
The geo-location of Shandong Province in China (the map was created with ArcGIS software, 10.2). The figure shows the geographical location of Shandong Province and the location is depicted on the map using the software ArcGIS10.2 (https://www.arcgis.com/features/index.html, ESRI, Inc., Redlands, CA, USA).

Shandong Province is located 34°25′–38°23′ N and 112°43′–114°36′E and it covers approximately156700 square kilometers with 97.3 million people by the end of 2010. Figure 7 shows Shandong Province 17 cities located on the east coast of China. It has a semi-humid monsoonal climate with hot and rainy summer, and cold and dry winter. The annual average precipitation is around 550–950 mm and the annual average temperature is 11 °C–14 °C.The east of Shandong Province expands into the sea, the central and southern areas are surrounded with mountains and hills, and the northern and western regions belongs to the North China Plain. Jinan serves as the capital and cultural center of the province, while the coastal areas have more advanced economics.

### Data collection

Daily disease data were obtained from the Shandong Disease Reporting Information System (SDRIS) in Shandong Center for Disease Control and Prevention. The diagnosis of mumps was made according to the criteria established in the “Diagnostic criteria for mumps” published by the Chinese Ministry of Health. To clean the data and rule out bacteroidal infection, we checked whether the cases had unilateral or bilateral nonsuppurative parotid or other salivary swelling without apparent cause. The notified mumps cases were the ones who had no increase in white blood count by exclusion of infection. During the study period, the diagnostic standard for mumps was consistent. All the cases were diagnosed by trained professionals in qualified hospitals. To prevent infectious disease outbreaks, China has established a strict management system for reporting notifiable and communicable diseases. For mumps, public health agencies should report to the internet within 24 hours. If they do not follow this, they should send out the infectious disease report cards within 24 hours. Professionals fill out the infectious disease report cards, and then these cards are collected by experienced reporters. Before analyzing the data, data cleaning was undertaken, including removing duplicate results according to the identification card numbers. This process ensures the timeliness, accuracy, integrity of the data.

### Descriptive and geographical analysis

To better understand and visualize the geographical pattern, we first utilized descriptive and geographical analyses respectively. The spatial distribution of mumps incidence is described using geographical information system. There are several reasons why we have not adjusted factors such as age, sex, etc. First, the population age and gender structures are little changed, so they have smaller influence on the overall trend of mumps. And this is a grouped level analysis, not easy to adjust the individual factors. Secondly, in regular epidemiological studies, if we want to compare the incidence of different counties, we should adjust factors, according to the united standard. But the information in the paper about the annual rate is used to describe the spatial, temporal and population distribution of mumps. Lastly, the main goal was to reveal evidence of mumps spatial heterogeneity. The incidence rates of mumps were computed at county level and colored with different colors in the map using the software ArcGIS10.2 (ESRI, Redlands, CA, USA). Geographical data was accessed from digital maps from the National Fundamental Geographic Information System, China. The disease spatial distribution map shows the incidence of mumps and it can provide the guidance for further study. However, the distribution map can only show the distribution of actual values, but not the comparison ratio. This hazard ratio (Geoda software calls these values excess hazard ratios) is defined as the ratio of the incidence in the specific county over the average incidence in Shandong Province^20^,^27^. To show the ratio level over the whole area, the hazard ratio was computed and visualized as the hazard ratio map. The hazard ratio can be calculated using the formula: the incidence in one county / the average incidence in whole province. The county whose hazard ratio value is less than 1 declares it has lower incidences than expected, while the one whose hazard ratio value is greater than 1 implies it has higher incidences than expected.

### Spatial autocorrelation analysis

Spatial autocorrelation is a method to detect the correlations from spatial domain and it includes two categories, one is the global spatial autocorrelation which detects the distribution feature from an overview aspect, the other is the local spatial autocorrelation which recovers the feature in a more local region^28^,^29^,^30^. It is built in GeoDa. a popular open source software. The Moran’s I was used to examine the global and local level of spatial autocorrelation, and to determine locations of hotspots. We assume that the risk of everyone in getting infected is the same and the spread process can be regarded as a classic statistical model–poisson distribution. The null hypothesis assumes that the disease is distributed randomly in Shandong Province. Spatial weight coefficient is calculated according to spatial adjacency created by queen contiguity rule. The spatial weight coefficient is used to determine the relevant space, and then we can examine the statistical significance according to the standardized statistics. The range of Moran’s I is -1 to 1. If Moran’s I is statistically greater than zero, it shows a positive spatial autocorrelation. If Moran’s I is statistically less than zero, there exists a negative spatial autocorrelation.

Local indicators of spatial autocorrelation (LISA) reveal four different spatial patterns as high-high, low-low, high-low and low-high. High-high means that high-incidence regions surrounded by high-incidence regions. Low-low means low-incidence regions surrounded by low-incidence regions. High-low means high-incidence regions surrounded by low-incidence regions and low-high means low-incidence regions surrounded by high-incidence regions. The number of permutation test was set to 999 and the significance level was set as 0.05.

### Scan statistics

Next, we aim to extract the spatial-temporal clusters of mumps. The space-time scan proposed by Kulldorff is a typical technique to detect the clusters for disease outbreak detection and has been integrated in SaTScan^TM^ v9.4. In SaTScan, it includes purely space scan statistics, space-time scan statistics and purely temporal scan statistics. The purely spatial scan statistic imposes a circular window on the map. The space-time scan statistic is defined by a cylindrical window with a circular geographic base and the height of window corresponds to time^31^. The null hypothesis is that the disease is distributed randomly, while the alternative hypothesis is that the incidence of the disease within the window increases compared to outside. The hypothesis-testing statistic of the model is log likelihood ratio (LRR), which is calculated by Monte Carlo randomization method. Scan window with the largest LRR is treated as primary cluster and the other areas with the significant statistical LLR are defined as the secondary clusters. In this paper, the maximum radius was set as 15% of the total population, and the maximum time scan period was set as 50% of the overall study period. Scientific research suggests that the radius of scan window is less than 30%, the length of the true cluster should be less than 10%-15% of the total number of regions and the number of the cluster is less than 75% of the total number of regions^32^,^33^. These settings can detect a modest number and a suitable radius of clusters with high accuracy and stable result. We have tested the radius from 15% to 30% and from the result, the radius with 15% performs best and we chose 15% as the radius. And we also chose maximum period set as 50% of the total study period based on previous research^34^. The ratio of the incidence inside and outside the window is RR. For each potential existing spatial cluster, p value of LLR is obtained from Monte Carlo simulation^35^. The number of permutations and the significance level were set as 999 and 0.05 respectively.

## Additional Information

**How to cite this article:** Li, R. *et al*. Epidemiological Characteristics and Spatial-Temporal Clusters of Mumps Disease in Shandong Province, China, 2005–2014. *Sci. Rep.* **7**, 46328; doi: 10.1038/srep46328 (2017).

**Publisher's note:** Springer Nature remains neutral with regard to jurisdictional claims in published maps and institutional affiliations.

## Figures and Tables

**Table 1:** Demographic characteristics of patients with mumps in Shandong Province, China, 2005–2014.

Variables	Case number	Percentage (%)
Gender		
Male	76433	66.04
Female	39312	33.96
Age		
≦4	16814	14.53
5–9	47215	40.79
10–20	40326	34.84
21–30	5709	4.93
31–40	3571	3.09
41–50	1246	1.08
51–60	550	0.48
>60	314	0.27
Seasons		
Spring	37231	32.17
Summer	32920	28.44
Autumn	17815	15.39
Winter	27779	24.00
**Total**	115745	100

**Table 2:** The global spatial autocorrelation of mumps in Shandong Province, 2005–2014.

Year	Moran’s I	Mean	Sd	Z Score	P Value
2005	0.151811	−0.0064	0.0397	3.9867	0.005
2006	0.461606	−0.0059	0.0493	9.4893	0.001
2007	0.328159	−0.0087	0.0543	6.2024	0.001
2008	0.365535	−0.0047	0.0534	6.9370	0.001
2009	0.355728	−0.0036	0.0528	6.8030	0.001
2010	0.395776	−0.0062	0.0517	6.9335	0.001
2011	0.35197	−0.0046	0.0527	6.7653	0.001
2012	0.247423	−0.0070	0.0517	4.9175	0.001
2013	0.2025	−0.0065	0.0489	4.2705	0.003
2014	0.208732	−0.0076	0.0512	4.2222	0.001

**Table 3:** The local spatial autocorrelation of mumps in Shandong Province, 2005–2014.

Town	LISA_I	P-value	Clusters
Lixia District	0.591132	0.049	Hotspot
Shizhong District in Jinan City	0.302213	0.017	Hotspot
Shinan District	1.207407	0.016	Hotspot
Shibei District	3.119204	0.014	Hotspot
Sifang District	4.914925	0.001	Hotspot
Laoshan District	2.113898	0.004	Hotspot
Licang District	4.279192	0.018	Hotspot
Chengyang District	0.347652	0.045	Hotspot
Linzi District	0.969057	0.01	Hotspot
Gaoqing County	0.309739	0.032	Hotspot
Dongying District	0.791019	0.007	Hotspot
Shizhong District in Jining City	6.801319	0.024	Hotspot
Boxing County	1.651034	0.002	Hotspot

**Table 4:** Space-time clusters with higher incidence in Shandong Province, China from 2005–2014.

Cluster type	Time frame	Cluster areas(n)	Observed cases	Expected cases	Radius	Relative risk	*p* value
Most likely	2012–2013	5	6333	699.82	34.68	9.48	<0.0001
Secondary	2008–2011	2	6350	1104.47	4.88	6.00	<0.0001
2nd Secondary	2012–2013	21	10736	3639.79	76.00	3.13	<0.0001
3rd Secondary	2005–2005	1	1442	81.63	0.00	17.86	<0.0001
4th Secondary	2011–2014	5	5053	1475.12	23.28	3.53	<0.0001
5th Secondary	2009–2013	8	6876	3206.19	58.92	2.21	<0.0001
6th Secondary	2012–2013	13	4298	2503.22	77.76	1.74	<0.0001
7th Secondary	2013–2014	3	1261	623.13	37.03	2.03	<0.0001
8th Secondary	2008–2008	3	736	297.34	40.86	2.48	<0.0001
9th Secondary	2012–2013	12	4189	2979.08	119.16	1.42	<0.0001
